# Antioxidant Capacities of Fractions of Bamboo Shaving Extract and Their Antioxidant Components

**DOI:** 10.3390/molecules21080996

**Published:** 2016-07-30

**Authors:** Jinyan Gong, Jun Huang, Gongnian Xiao, Feng Chen, Bolim Lee, Qing Ge, Yuru You, Shiwang Liu, Ying Zhang

**Affiliations:** 1 Zhejiang Provincial Collaborative Innovation Center of Agricultural Biological Resources Biochemical Manufacturing, Zhejiang Provincial Key Lab for Chem and Bio Processing Technology of Farm Produces, School of Biological and Chemical Engineering, Zhejiang University of Science and Technology, Hangzhou 310023, China; hjunlzr@163.com (J.H.); xiaogongnian@126.com (G.X.); gq0318@163.com (Q.G.); youyuru0130@163.com (Y.Y.); shiwangliu@163.com(S.L.); 2 College of Biosystems Engineering and Food Science, Zhejiang University, Hangzhou 310058, China; yzhang@zju.edu.cn; 3 Department of Food, Nutrition and Packaging Sciences, Clemson University, Clemson, SC 29634, USA; fchen@clemson.edu (F.C.); boliml@g.clemson.edu (B.L.)

**Keywords:** bamboo shavings extract, antioxidants, free-radical scavenging activity, flavonoids, phenolic acids

## Abstract

This research was conducted for evaluation of antioxidant activities of four fractions from bamboo shavings extract (BSE) and their antioxidant components. The antioxidant capacities of BSE and four fractions on ABTS, DPPH, FRAP and total antioxidant capacity assays exhibited the following descending order: DF > *n*-butanol fraction (BF) > BSE ≈ ethyl acetate fraction (AF) > water fraction (WF). Among the identified phenolic compounds, caffeic acid exhibited the highest antioxidant capacities on DPPH, FRAP and total antioxidant capacity assays. An extremely significant positive correlation between the antioxidant activities with the contents of total flavonoids, total phenolic acids, or total phenolics was observed in this study. The result indicated that the bamboo shaving extract and its solvent fractions could act as natural antioxidants in light of their potent antioxidant activities.

## 1. Introduction

It is well known that oxidative damages caused by reactive oxygen species (ROS), such as superoxide (O_2_^−^), hydroxyl (OH•) and peroxyl (ROO•) radicals, in the human body may trigger various chronic diseases such as cancer, aging, atherosclerosis, inflammations, etc. [1,2]. Even though organisms have self-antioxidant defense systems that are able to repair oxidative damages and protect themselves from free radicals, it is often insufficient to capture all free radicals, or, in some cases, there may be a risk that the antioxidant mechanisms are overrun (i.e., the cellular redox potential shifts toward oxidative stress) [3]. Therefore, significant researches have been conducted in an effort to explore natural antioxidants to prevent and treat chronic diseases such as cardiovascular disease, cancer, etc. Meanwhile, some secondary metabolites, such as phenolic acids, flavonoids, steroids, alkaloids, tannins, terpenoids and terpenes, have deemed some plant extracts as real alternative medicines for arthritis, diabetes, ulcers, infections, asthma, hypertension, central nervous system disorders, laryngitis and liver disorders, whose antioxidant, anxiolytic, antiradiation, antibacterial, anti-inflammatory, antidepressant, antihypertensive, analgesic and anticancer properties have attracted more and more attention [4,5,6,7,8].

**Figure 1:**
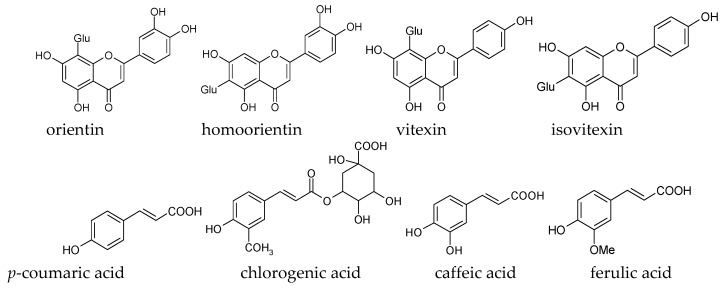
Chemical structure of the studied four flavone *C*-glucosides and four phenolic acids in bamboo shavings extract (BSE). Glu: glucose.

Bamboo shavings extract (BSE) has been used in traditional Chinese medicines for treatment of stomach-ache, diarrhea or vomiting, chest diaphragm inflammation, restlessness, excessive thirst, etc., which had been described in the Chinese Materia Medica for over 1000 years [9,10,11]. Previous studies indicated that the BSE is a safe product in light of its low toxicity [10], and has effective capacity in scavenging DPPH free radicals and other ROS radicals. Our preliminary research also showed that BSE is rich in natural bioactive chemicals, including chlorogenic acid, caffeic acid, ferulic acid, *p*-coumaric acid, orientin, homoorientin, vitexin, isovitexin, etc. (shown in Figure 1) [11]. However, no systematic study has been conducted to examine the relationship between the functional components of BSE and its antioxidant capacities.

Therefore, the present study was designed to study the antioxidant activities of the BSE and its solvent fractions, and evaluate the activities of their inherent antioxidant constituents. It is expected that this research will provide more evidence and insights into the bioactivities of bamboo shavings in an effort to better utilize it as a potential health benefiting natural product or functional food ingredient, as well as the possibility of using the BSE as a source of low-cost natural antioxidants.

## 2. Results

### 2.1. Total Phenolics and Total Flavonoids Contents

It has been recognized that, in many cases, the antioxidant activities of plant extracts are correlated with their total phenolics content. These phenomena were often ascribed to the natural flavonoids and other phenolic compounds in the extracts, which are usually very effective antioxidants [12]. 

From our previous studies, some phenolics and flavonoids have been isolated from the BSE [11]. In this study, the bamboo shavings powder were first extracted by CO_2_ SFE, followed by ethanol-water (30:70, *v*/*v*) extraction, to get the fat-free BSE, which was then resuspended in warm water and extracted sequentially by diethyl ether, ethyl acetate, *n*-butanol and water to obtain the DF, AF, BF and WF fractions, respectively. The non-polar and weak polar compounds in the original BSE after the ethanol-water extraction were easily dissolved in diethyl ether, while ethyl acetate was used to extract medium polar compounds and glycosides. Polar compounds like polypeptides and sugars remained after the aforementioned solvent extractions and were more likely to be extracted by *n*-butanol and water.

Table 1 shows the extraction yield (EY), contents of total phenolics (TP) and total flavonoids (TF) of each fraction from the bamboo shavings. The extraction yield of all fractions varied from 53.8% to 5.5%. Among all the fractions, the BF obtained the highest extraction yield (53.8% ± 5.1%) while the AF had the lowest yield (5.5% ± 0.5%). This phenomenon was ascribed to the polarity of the extraction solvents and the extraction procedures upon the bamboo shavings, which was initially under a CO_2_ SFE extraction followed by polar ethanol–water (30:70, *v*/*v*) extraction. Therefore, the majority of the extracted compounds in the original BSE extract were medium polar and polar chemicals. In this context, the yield of all the fractions is presented in the following order: BF > WF > BSE > DF > AF. The low extraction yield of AF is obviously due to the low solubility of some components in the original BSE.

The total phenolics (TP) contents of all fractions, based on the same dry weight regardless of their recovery yields from bamboo shavings, varied from 28.4% to 71.7%. The highest content of TP was measured at 71.7% ± 3.8% in the DF, whereas the lowest content was found at 28.4% ± 1.4% in the WF (Table 1). The TP values of the BSE fractions were found to be arranged in the following descending order: DF > AF > BF > BSE > WF. This result was consistent with the fact that the DF fraction was rich in some non-polar and weak polar phenolic compounds, such as *p*-coumaric acid, which accounted for 26.32 ± 0.23 mg/g (Table 2).

In contrast, the contents of total flavonoids (TF) of all the fractions from bamboo shavings varied from 10.2% to 30.6%. The highest TF value was observed in the DF (30.6% ± 2.1%), while the lowest content was found in the WF (10.2% ± 0.5%). The TF values of the BSE and its fractions are listed in the following descending order: DF > BF > AF > BSE > WF. This order is slightly different from that of the TP values. Nevertheless, the result was consistent of the fact that the DF fraction was rich in some non-polar and weak polar flavonoids, such as tricin, from bamboo shavings, which has been reported in our previous preliminary research [10,13].

### 2.2. Contents of Four Flavone C-Glucosides and Four Phenolic Acids 

The main functional components of BSE included four flavone C-glycosides, including orientin, homoorientin, vitexin, and isovitexin, and four phenolic acids, including chlorogenic acid, caffeic acid, ferulic acid, and *p*-coumaric acid. All of these compounds have been isolated and characterized in our previous preliminary research [11]. However, these bioactive components have not been investigated in regards of their distributions in the BSE fractions. In this context, the aforementioned four fractions of BSE were analyzed by a reverse-phase HPLC, as shown in (Supplementary material Figure S1), while the characteristic compounds are listed in Table 2.

From Table 2, the highest contents of chlorogenic acid and caffeic acid were observed in the BF fraction (0.72 ± 0.02 mg/g DW and 0.22 ± 0.01 mg/g DW, respectively); In contrast, the DF had the highest content of *p*-coumaric acid (26.32 ± 0.23 mg/g DW), while AF had the highest content of ferulic acid (0.46 ± 0.01 mg/g DW). In regards of the flavone *C*-glucosides in four fractions, the AF fraction was found to have the highest amounts of homoorientin (1.56 ± 0.02 mg/g DW), orientin (1.90 ± 0.04 mg/g DW) and isovitexin (0.98 ± 0.02 mg/g DW), while the content of vitexin was lower in every fraction than in the BSE (0.67 ± 0.04 mg/g DW). 

### 2.3. Antioxidant Activity

#### 2.3.1. ABTS Radical Scavenging Activity

**Figure 2:**
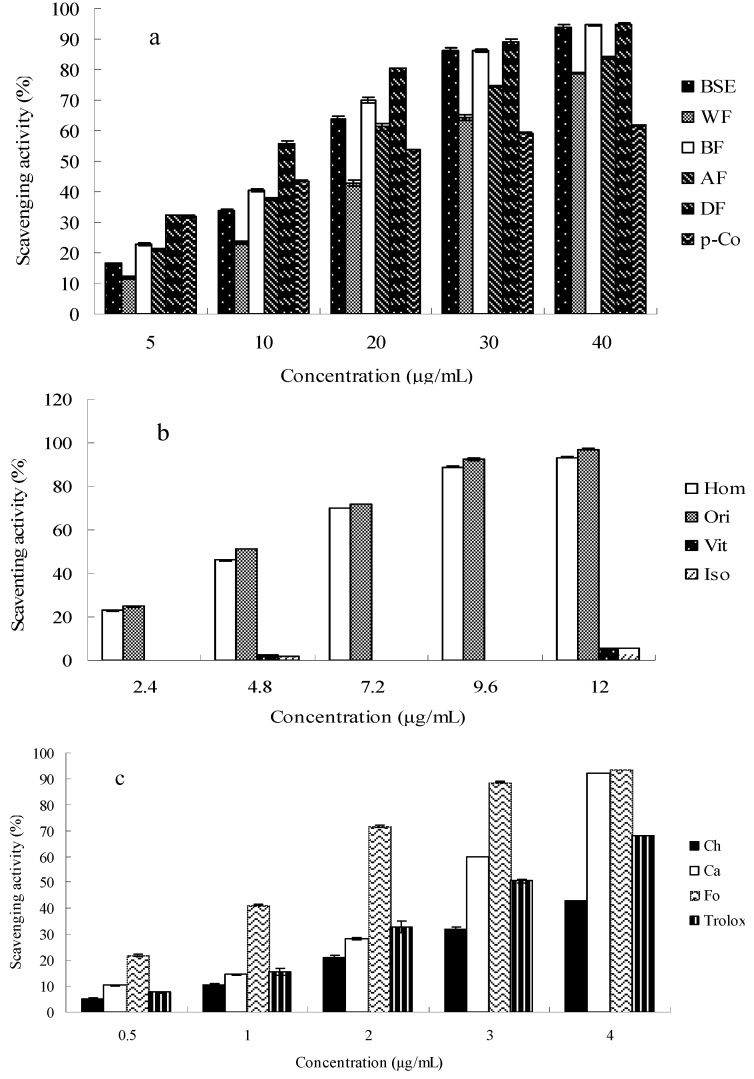
Scavenging activities of fractions (**a**) four fractions with *p*-coumaric acid and characteristic compounds (**b**) four flavone *C*-glucosides, (**c**) three phenolic acids with Trolox of bamboo shavings extract on stable ABTS radical; BSE, bamboo shavings extract; DF, Diethyl ether fraction; AF, acetic ether fraction; BF, *n*-butanol fraction; WF, water fraction; Hom, homoorientin; Ori, orientin; Vit, vitexin; Iso, isovitexin; Ch, chlorogenic acid; Ca, caffeic acid; Fo, ferulic acid; *p*-Co, *p*-coumaric acid.

ABTS^●+^ is a synthetic radical that can be used to assess the scavenging activity of both polar and non-polar antioxidant chemicals [14]. Therefore, this assay was suitable to further assess the BSE fractions and their characteristic compounds against the synthetic ABTS^●+^ free radical. Figure 2 shows the percentages of scavenging ABTS^●+^ radicals by all the fractions and individual bioactive compounds at different concentrations, which exhibit good correlations with their concentrations.

The BSE was able to scavenge the ABTS^●+^ radicals in a concentration dependent manner (Figure 2a). Although the BSE showed a lower scavenging ability with an IC_50_ value of 2.97 ± 0.08 μg/mL against the ABTS^●+^ than Trolox (a derivative of Vitamin E), it still showed a 93.76% scavenging ability against the ABTS^●+^ at the concentration of 40 μg/mL (Figure 2a). Similarly, the DF fraction at the concentration of 40 μg/mL showed a scavenging ability at 94.95%, which was the strongest ABTS^●+^ scavenging ability among all the fractions.

Among the phenolic acids, ferulic acid (IC_50_ 7.21 ± 0.31 µmol/L) and caffeic acid (IC_50_ 9.64 ± 0.28 µmol/L) exhibited stronger ABTS^●+^ scavenging abilities, of which the inhibitive percentages were 93.74% ± 0.23% and 92.13% ± 0.61% at 4 μg/mL, respectively. Besides, both of them were stronger than that of Trolox (68.12% ± 0.76%, IC_50_ 11.87 ± 0.32 µmol/L) at 4 μg/mL. In contrast, *p*-coumaric acid had the lowest value with the IC_50_ value of 101.12 ± 1.10 µmol/L (Figure 2a,b and Table 3). Among the flavone C-glycosides, the IC_50_ values of the ABTS^●+^ scavenging abilities of orientin and homoorientin were 10.86 ± 0.25 and 11.53 ± 0.45 µmol/L, respectively, corresponding to their inhibitive percentages at 97.05% ± 0.31% and 93.66% ± 0.08%, at their concentration of 12 μg/mL, respectively, which was much higher than vitexin (5.77% ± 0.31%) and isovitexin (5.69% ± 0.18%) at the same concentration (Figure 2c and Table 3).

#### 2.3.2. DPPH Radical Scavenging Activity

The antioxidant ability of the fractions from bamboo shavings to quench reactive species by hydrogen donation was measured by the DPPH free radical scavenging activity assay. Since the DPPH is a relatively stable free radical, it can accept an electron or hydrogen radical to become a stable diamagnetic molecule, which is widely used to investigate the radical scavenging activity. 

**Figure 3:**
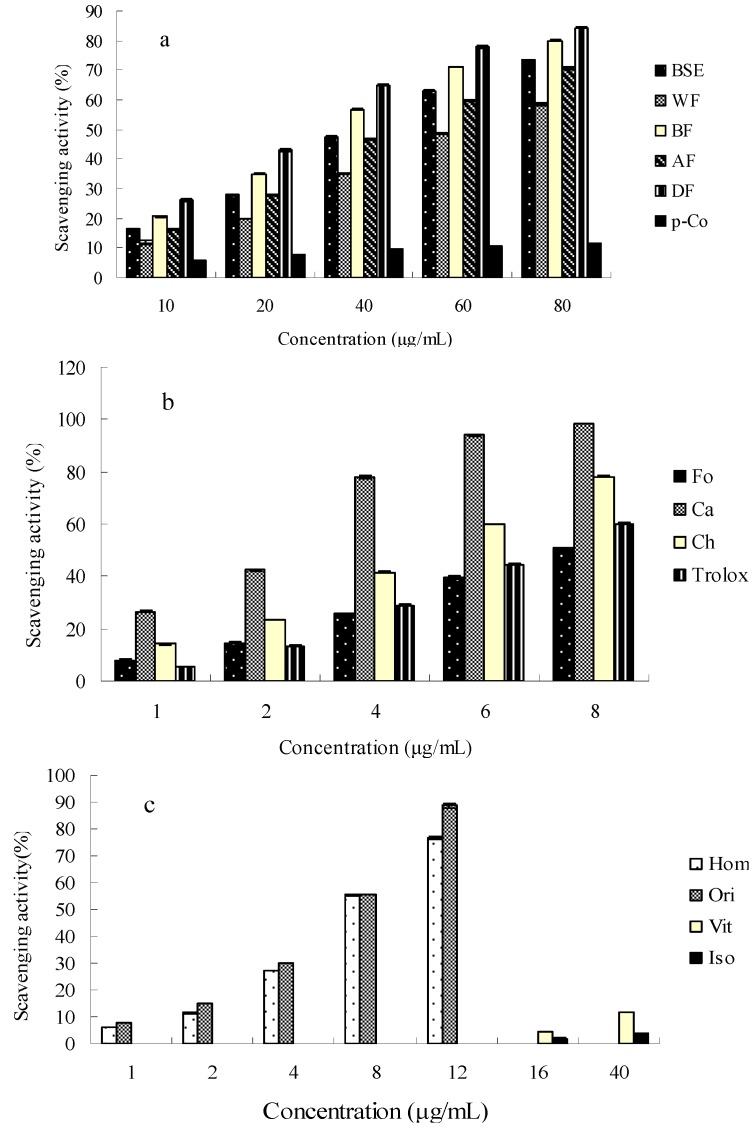
Scavenging activities of fractions (**a**) four fractions with *p*-coumaric acid and characteristic compounds (**b**) four flavone *C*-glucosides, (**c**) three phenolic acids with Trolox of bamboo shavings extract on stable DPPH radical; BSE, bamboo shavings extract; DF, Diethyl ether fraction; AF, acetic ether fraction; BF, *n*-butanol fraction; WF, water fraction; Hom, homoorientin; Ori, orientin; Vit, vitexin; Iso, isovitexin; Ch, chlorogenic acid; Ca, caffeic acid;Fo, ferulic acid; *p*-Co, *p*-coumaric acid.

The DPPH free radical scavenging activity of all the fractions from bamboo shavings increased along with the increasing concentration of the fractions (Figure 3a–c). Figure 3 shows that the power of all the extracts and its compounds to scavenge the DPPH free radicals was correlated with their concentrations. The DF possessed the highest scavenging activity (83.39% ± 0.28%) among all the fractions at 80 μg/mL, while the Trolox showed a scavenging activity of 60.10% ± 0.30% at 8 μg/mL (shown in Figure 3). As shown in Table 3, the IC_50_ values corresponding to the DPPH free radical scavenging activity were found to be 42.09 ± 2.16 μg/mL, 59.20 ± 3.13 μg/mL, 33.95 ± 2.24 μg/mL, 42.99 ± 3.58 μg/mL, 27.42 ± 1.81 μg/mL and 6.70 ± 0.16 μg/mL for the original BSE, WF, BF, AF, DF and Trolox, respectively. 

Among the phenolic acids, caffeic acid had the strongest DPPH^●^ scavenging ability with an IC_50_ value of 8.41 ± 0.81 µmol/L, which is lower than that of the positive control (Trolox, IC_50_ 26.77 ± 0.64 µmol/L); chlorogenic acid (IC_50_ 13.89 ± 0.96 µmol/L) also had a stronger DPPH^●^ scavenging ability than the Trolox. Meanwhile, *p*-coumaric acid showed the lowest DPPH^●^ scavenging ability with an IC_50_ 2304.91 ± 76.82 µmol/L. Among the flavone C-glycosides at their concentrations at 12 μg/mL, the DPPH^●^ scavenging capability of orientin and homoorientin was 88.79% ± 0.08% and 76.48% ± 0.06%, respectively, with the corresponding IC_50_ values at 15.30 ± 0.40 µmol/L and 17.06 ± 0.56 µmol/L, respectively, which were much stronger than vitexin (11.81% ± 0.420%) and isovitexin (3.83% ± 0.17%) at 40 μg/mL.

#### 2.3.3. Ferric-Reducing Antioxidant Power (FRAP)

FRAP measures the antioxidant capacity of any reducing substance in the reaction medium. In the present study, as shown in Table 3, the DF exhibited the strongest reducing power (180.39 ± 2.09 mg TEAC/g DW) among all the fractions, while the original BSE showed 113.07 ± 4.99 mg TEAC/g DW, BF with 127.72 ± 0.52 mg TEAC/g DW, AF with 116.56 ± 2.18 mg TEAC/g DW, and WF with 63.54 ± 1.48 mg TEAC/g DW. In regards of the phenolics, caffeic acid had the strongest reducing power (4312.72 ± 72.03 mg TEAC/mmol DW), which is even stronger than Trolox (999.48 ± 52.22 mg TEAC/mmol DW). Others were weaker than Trolox. *p*-Coumaric acid had the lowest reducing power of 1262.50 ± 12.73 mg TEAC/mmol DW among the four phenolic acids; orientin had the strongest reducing power (1578.22 ± 26.67 mg TEAC/mmol DW) among the four flavonoids, followed by homoorientin (1442.08 ± 24.04 mg TEAC/mmolg DW) and vitexin (54.58 ± 3.19 mg TEAC/mmol DW). Isovitexin showed the lowest reducing power with the value of 37.63 ± 2.42 mmol TEAC/g DW.

#### 2.3.4. Total Antioxidant Capacity (TAC)

As shown in Table 3, the DF exhibited the greatest total antioxidant capacity (35.26 ± 1.00 U/mg DW), followed by BF 26.65 ± 0.50 U/mg DW, BSE 18.36 ± 0.68 U/mg DW, AF 16.25 ± 0.56 mg U/mg DW, while WF had the lowest value (12.19 ± 0.50 U/mg DW). 

Regarding the phenolics, caffeic acid had the strongest total antioxidant capacity (1374.03 ± 11.50 U/µmol DW), followed by chlorogenic acid (703.56 ± 2.79 U/µmol DW), ferulic acid 684.54 ± 5.15 U/µmol DW, Trolox (684.33 ± 11.71 U/µmol DW) and *p*-coumaric acid 21.69 ± 7.49 U/µmol DW; In comparison, orientin exhibited the strongest total antioxidant capacity (76.83 ± 0.82 U/µmol DW), followed by homoorientin (75.38 ± 1.12 U/µmol DW), vitexin (11.29 ± 1.57 U/µmol DW) and isovitexin (10.52 ± 0.76 U/µmol DW).

#### 2.3.5. Correlation of Total Flavonoids, Total Phenolics, and Total Phenolic Acids with Antioxidant Assay

The antioxidant activities of flavonoids and phenolic acids are determined by their chemical structures, particularly the hydroxylation status of their aromatic rings. It has been shown that antioxidant activity is related to flavonoids containing a phenol B ring. 3′,4′-catechol, the C2–C3 double bond configured 4-oxo, 5-OH, and arrangements on the flavan skeleton are the main structural determinants of antioxidant activities of flavonoids [14,15,16]. The presence of the 3,4-position of dihydroxylation on the phenolic ring and additional conjugation in the propenoic side chain give higher competitive antioxidant activity for phenolic acids, which might facilitate the electron delocalization, between the aromatic ring and propenoic group, by resonance [15,17].

Impei et al. (2015) [18] observed a significant correlation in the extracts of *Vitis vinifera* L. between the metabolite concentration and antioxidant effects. Yao et al. (2010) [19] have demonstrated a high correlation between the content of total phenolic acids, total flavonoids and total phenolics with their antioxidant capacity in 11 celery cultivars. The results obtained in our study (Table 4) showed that the contents of total flavonoids, total phenolics and total phenolic acids significantly correlated with the values of ABTS (IC_50_), DPPH (IC_50_), FRAP and total antioxidant capacity assay, which suggests that contents of total flavonoids, total phenolics and total phenolic acids all contributed to the antioxidant capacities of bamboo shavings fractions. DF had the highest antioxidant activity, which might partially be due to its higher levels of total flavonoids, total phenolics and total phenolic acids. 

## 3. Discussion

Bamboo shavings extract (BSE) contains many bioactive components, including phenolic acids and flavonoids, such as caffeic acid, chlorogenic acid, ferulic acid, *p*-coumaric acid, orientin, homoorientin, vitexin and isovitexin, which are all natural antioxidants. After the BSE was extracted by CO_2_ SFE and partitioned sequentially with diethyl ether, ethyl acetate, *n*-butanol and water, it was found that *p*-coumaric acid was subject to dissolution in diethyl ether; ferulic acid, orientin, homoorientin and isovitexin were more easily dissolved in ethyl acetate, while caffeic acid and chlorogenic acid were less easily dissolved in n-butanol. As a result, the WF, partially due to the lack of phenolic acids and flavonoids, had a lower total antioxidant activity than AF and BF. However, the higher concentration of weak polar compounds, such as methoxyflavones and flavonoid aglucone like tricin in DF, than those in other fractions and BSE, might have led to the stronger antioxidant activities in all the aforementioned in vitro antioxidant models [13]. 

Although the contents of phenolic acids and flavonoids were often used as important biomarkers of antioxidant capacity, it was also found that the antioxidant activities of the BSE and the other four fractions were not in the same descending order in the different in vitro models. 

The diethyl ether (DF) fraction possessed the strongest antioxidant activities in all antioxidant tests. The antioxidant and free-radical scavenging activities of the BSE and its four fractions may be attributed to their chemical composition, especially phenolic acids and flavonoids. The biological activities of phenolic compounds strongly depend on their chemical structures and spatial structures of various moieties on the molecule [18]. Even minor changes in their chemical structures (e.g., position of substituted group on flavan skeleton) can have a remarkable impact on their biological activities [20]. Biological activities of flavonoid are frequently linked to their antioxidant properties. For example, redox activity contributes significantly to free radical defense [21]. 

Orientin and homoorientin are chemicals isomers, so are the isovitexin and vitexin. Orientin has an additional 3′-OH over vitexin, as does homoorientin over isovitexin. Besides, orientin and vitexin have their bound sugar moieties in the C8 position, while homoorientin and isovitexin link their conjugated sugars in the C6 position (Figure 1); Orientin and homoorientin had higher antioxidant activities than vitexin and isovitexin, respectively, which were ascribed to the existence of more hydroxyl groups, i.e., 3′,4′-OH in the B ring, while isovitexin and vitexin have only 4′-OH.

Flavonoids have shown various biological activities, particularly in light of their pharmaceutical functions. The presence of the sugar moiety significantly affects bioavailability, pharmacokinetic properties and most of the positive health effects of flavonoids, including antioxidant, anticancer, antiinflammatory and antibacterial actions [22]. In this study, homoorientin showed a weaker antioxidant activity than orientin, the same as that of isovitexin vs. vitexin. When the glucose is attached to homoorientin and isovitexin at C6, their structures seem linear, which causes more steric hindrance to affect the binding of radical reagent. In contrast, when orientin and vitexin have the glucose bound at C8, a corner of less than 90° will exist inside the flavonoid molecule and lead the three-dimensional structure of the flavonoid to look like a “V”. Molecules with a “V” structure are more easily trapped with radical reagent and exhibit higher stability [23]. This might explain the significant differences between their antioxidant activities owing to the differences in their stereo-structures. 

No matter the kind of in vitro antioxidant models—including the ABTS, DPPH radical scavenging activity, reducing power and total antioxidant capacity—were used in the aforementioned antioxidant tests, caffeic acid, chlorogenic acid and ferulic acid always exhibited stronger antioxidant activities than *p*-coumaric acid. This phenomenon was ascribed to existence of a 3,4-catechol or 4-OCH_3_ group that enhanced the ability to donate electrons [24]. 

## 4. Materials and Methods

### 4.1. Chemical Reagents and Materials

2,2-Diphenyl-1-picrylhydrazyl (DPPH) was purchased from Sigma-Aldrich (Steinheim, Germany). Folin-Ciocalteu reagent, 2,2′-azinobis-(3-ethylbenzothiazoline-6-sulphonic acid) diammonium salt (ABTS), 2,4,6-Tri (2′-pyridyl)-1,3,5-triazine (TPTZ) and 3-aminophthalhydrazide, chemical standards in HPLC-grade, such as rutin, gallic acid, *p*-coumaric acid, chlorogenic acid, caffeic acid and ferulic acid, were purchased from Sigma chemical Co. (St. Louis, MO, USA). Orientin, homoorientin, vitexin and isovitexin standards (HPLC-grade) were obtained from Extrasynthese Company Inc. (Lyon, France). Ultrapure water was prepared by the Milli-Q system (Millipore, Bedford, OH, USA). All solutions prepared for HPLC were passed through 0.45 μm nylon filters before use. All other chemicals were of analytical reagent grade.

### 4.2. Samples

The bamboo shavings extract (BSE) was prepared according to the procedures previously established by our research team [11]. In brief, 2000 g of the clean bamboo shavings powder (10–20 mesh) was extracted by 30% (*v*/*v*) ethanol solution for 1.5 h by a hot reflux method after a carbon dioxide (CO_2_) supercritical fluid extraction (SFE), and dried into the final product via a spray dry treatment. Then, 206 g of the BSE was resuspended in warm water and then partitioned sequentially with diethyl ether, ethyl acetate, *n*-butanol and water. The diethyl ether fraction (DF), ethyl acetate fraction (AF), *n*-butanol fraction (BF) and water fraction (WF) were collected respectively, and concentrated using a rotary flash evaporator (N-1200AV-W, Tokyo Rikakikai Co. Ltd., Tokyo, Japan) to remove the solvents, respectively. The respective residues were dissolved in water and then freeze dried. The original BSE and its four fractions (i.e., AF, BF, DF, and WF) were stored at 4 °C until analyses.

### 4.3. Total Phenolics Content and Total Flavonoids Content

The contents of total phenolics and total flavonoids of each fraction, based on the same amount of dry weight regardless of their respective recovery yields from the original BSE, were analyzed by the aluminum chloride and Folin-Ciocalteu colorimetric methods, using rutin [25] and gallic acid [26] as the standards, respectively.

### 4.4. Quantification of Compounds in BSE and Its Fractions

Isolated compounds from the BSE and its four fractions were determined based on the previous method [11]. In brief, all the samples were analyzed on a Waters 2695 HPLC instrument (Waters, Milford, MA, USA), which was equipped with a Luna C_18_ column (250 mm × 4.6 mm id, 5 μm) at 40 °C and detected at 330 nm. A gradient program relied on a mobile phase, consisting of solvent A (acetonitrile) and solvent B (1% acetic acid aqueous solution, *v*/*v*) with the following program: 15% A (15 min), 15%–40% A (10 min), 40% A (9 min), 40%–15% A (6 min). The flow rate of the mobile phase was controlled at 1.0 mL/min, while the injection volume was 10 μL. 

### 4.5. Analyses of Antioxidant Activities

#### 4.5.1. ABTS Radical Cation Scavenging Activity

The ABTS assay was tested using the method described before [14]. The ABTS was dissolved in water into a 7 mM concentration. ABTS^●+^ was produced by reacting the ABTS stock solution with 2.45 mM potassium persulfate, which was allowed to stand in the dark at room temperature for 12–16 h before use. Prior to the assay, the stock solution was diluted in ethanol (about 1:89 *v*/*v*) and equilibrated at room temperature to give an absorbance of 0.700 ± 0.02 in a 1 cm cuvette detected at 734 nm. The reaction mixture was prepared by mixing 3.9 mL of ABTS solution, and 0.1 mL of samples at different concentrations in a 50% (*v*/*v*) methanol solution. After a mild vortex, the samples were kept at room temperature for 6 min and measured at 734 nm to obtain their OD values. The scavenging percentage was calculated according to the following equation:

(1) ABTS scavenging capacity (%) = (1 − Asample/Acontrol) × 100

where A_control_ and A_sample_ refer to the spectrometric absorbances of the control, and the extract or standard, respectively. All tests were run in triplicate to obtain the mean value. Data were expressed as IC_50_ which correspond to concentration of extracts dry weight (µg/mL) or characteristic compounds (µmol/L) able to decrease the initial concentration of ABTS radical cation by 50%. 

#### 4.5.2. DPPH Radical Scavenging Activity

The DPPH free radical scavenging activity was determined according to a previous method with some modifications [26]. In brief, the reaction mixture was prepared by mixing 3.9 mL of 0.101 mM DPPH solution in ethanol, and 0.1 mL of samples at different concentrations in a 50% (*v*/*v*) methanol solution. After a strong vortex, the samples were kept at room temperature for 1 h and then measured at 517 nm to obtain their OD values. 

The scavenging percentage was calculated according to the following equation:

(2) DPPH scavenging capacity (%) = (1 − A_sample_/A_control_) × 100

where A_control_ is the absorbance of the control, and A_sample_ is the absorbance of the extract or standard. All tests were run in triplicate to get the mean value. The sample concentration of extracts dry weight (µg/mL) or characteristic compounds (µmol/L) providing 50% inhibition (IC_50_) was calculated as described in the ABTS assay.

#### 4.5.3. Ferric-Reducing Antioxidant Power (FRAP) Assay 

The reducing ability of the original BSE and its four fractions was determined by using the FRAP assay described by Shi et al. [26] with some modifications. The FRAP reagent was freshly prepared from 300 mM acetate buffer (pH 3.6), 10 mM TPTZ in 40 mM HCl and 20 mM FeCl_3_. All three solutions were mixed together in a ratio of 10:1:1 (*v*/*v*/*v*). An aliquot of 0.1 mL of the tested sample solution was mixed with 3.9 mL of the FRAP reagent. The absorption of the reaction mixture was measured at 593 nm after 10 min of incubation at 37 °C. The standard concentrations of Fe (II) were prepared for construction of the calibration curve. The reducing power was expressed as equivalent concentration. This parameter was defined as the concentration of the sample having a ferric-reducing ability equivalent to Trolox, and expressed as microgram of Trolox equivalents per gram or millimole of all the fractions (mg TEAC/g DW or mg TEAC/mmol DW) [27].

#### 4.5.4. Total Antioxidant Capacity

The total antioxidant capacity of all the fractions was determined using a commercial kit (Jiancheng Biological Engineering Institute, Nanjing, China). 

Total antioxidant capacity was determined according to a previous method with some modifications [28]. The result was calculated by the equation below:

(3) Total antioxidant capacity (U/mg DW or U/µmol DW) = (A_sample_ − A_control_) × *N* × *n*/0.3

where A_sample_ is the absorbance value of the sample; A_control_ is the absorbance value of the control; *N* is diluted number of the reaction system; *n* is the diluted number of the sample.

### 4.6. Statistical Analysis

Data were expressed as mean ± standard deviation of three replicated determinations. One-way analysis of variance was used to determine significant difference at *p* < 0.05 using SPSS 11 (SPSS Inc., Chicago, IL, USA).

## 5. Conclusions

Bamboo shavings are frequently used in traditional Chinese medicines, but have not been intensively explored for its health benefits. This study was designed to evaluate the antioxidant and free-radical scavenging activities of BSE extract and its solvent fractions by using four in vitro antioxidant models. The diethyl ether fraction (DF) showed the highest total phenolic and flavonoids contents, and the highest antioxidant and free-radical scavenging activities.

In addition, major inherent phenolics and flavonoids in the BSE and its fractions were determined, and measured against the same above mentioned four in vitro antioxidant models, which demonstrated that they were potent antioxidant compounds. Caffeic acid exhibited the highest antioxidant activities on DPPH free radical scavenging activity, reducing power and total antioxidant capacity among the phenolic acids, just like orientin among the flavonoids.

This study indicated that the BSE and its fractions or its inherent major bioactive compounds have great potential to scavenge free radicals. The wide range of antioxidant activities of the extracts indicates the potential of bamboo shavings as a source of natural antioxidants or nutraceuticals with potential application in reducing oxidative stress with consequent health benefits. However, further work is needed to identify other inherent characteristic compounds in the diethyl ether fraction and investigate their in vivo antioxidant efficacy.

## Figures and Tables

**Table 1:** Comparative analysis of extraction yield, total phenolic content and total flavonoids content of bamboo shavings fractions obtained using different solvents.

Index	BSE	DF	AF	BF	WF
TP (%)	42.1 ± 1.7	71.7 ± 3.8	64.2 ± 2.9	55.0 ± 2.8	28.4 ± 1.4
TF (%)	15.9 ± 1.6	30.6 ± 2.1	20.7 ± 1.2	21.0 ± 0.8	10.2 ± 0.5
EY (%)	10.3 ± 1.2	9.2 ± 0.9	5.5 ± 0.5	53.8 ± 2.1	13.0 ± 0.6

Values are mean ± standard deviation of three replicate analyses. BSE, bamboo shavings extract; DF, Diethyl ether fraction; AF, acetic ether fraction; BF, *n*-butanol fraction; WF, water fraction; TP, total phenolic content; TF, total flavonoids content.; EY, Extraction yield.

**Table 2:** The contents of flavone *C*-glucosides and phenolic acids in BSE and fractions (mg/g DW).

Extracts	Chlorogenic Acid	Caffeic Acid	*p*-Coumaric Acid	Ferulic Acid	Homoorientin	Isovitexin	Orientin	Vitexin	Total Phenolic Acids
BSE	0.67 ± 0.02	0.10 ± 0.01	6.80 ± 0.15	0.18 ± 0.02	1.35 ± 0.05	0.91 ± 0.05	0.58 ± 0.06	0.67 ± 0.04	11.26 ± 0.04
DF	Nd	Nd	26.32 ± 0.23	0.05 ± 0.01	Nd	0.10 ± 0.02	0.04 ± 0.01	0.07 ± 0.01	26.58 ± 0.22
AF	0.39 ± 0.01	0.21 ± 0.01	2.70 ± 0.03	0.46 ± 0.01	1.56 ± 0.02	1.90 ± 0.04	0.35 ± 0.01	0.98 ± 0.02	7.47 ± 0.03
BF	0.72 ± 0.02	0.22 ± 0.01	4.80 ± 0.04	0.18 ± 0.01	0.62 ± 0.04	0.93 ± 0.04	0.50 ± 0.03	0.27 ± 0.02	8.24 ± 0.03
WF	0.56 ± 0.02	Nd	Nd	Nd	Nd	Nd	Nd	Nd	0.56 ± 0.02

Values are mean ± standard deviation of three replicate analyses. The contents of flavone *C*-glucosides and phenolic acids were calculated based on the dry weights of BSE and all fractions. BSE, bamboo shavings extract; DF, Diethyl ether fraction; AF, acetic ether fraction; BF, *n*-butanol fraction; WF, water fraction; TP, total phenolic content; TF, total flavonoids content.; EY, Extraction yield. Nd: not detected.

**Table 3:** Antioxidant and free-radical scavenging activities of all the fractions and the characteristic compounds of bamboo shavings.

Objects	IC_50_		Reducing Power	Total Antioxidant Capacity
	ABTS	DPPH		
BSE *	15.47 ± 0.15	42.09 ± 2.16	113.07 ± 4.99	18.36 ± 0.68
WF *	23.41 ± 0.25	59.20 ± 3.13	63.54 ± 1.48	12.19 ± 0.50
BF *	13.49 ± 0.20	33.95 ± 2.24	127.72 ± 0.52	26.65 ± 0.50
AF *	15.49 ± 0.13	42.99 ± 3.58	116.56 ± 2.18	16.25 ± 0.56
DF *	9.70 ± 0.08	27.42 ± 1.81	180.39 ± 2.09	35.26 ± 1.00
Chlorogenic acid **	13.15 ± 0.14	13.89 ± 0.96	863.57 ± 16.45	703.56 ± 2.79
Caffeic acid **	9.64 ± 0.28	8.41 ± 0.81	4312.72 ± 72.03	1374.03 ± 11.50
*p*-Coumaric acid **	101.12 ± 1.10	2304.91 ± 76.82	1262.50 ± 12.73	21.69 ± 7.49
Ferulic acid **	7.21 ± 0.31	40.32 ± 2.36	3244.35 ± 17.92	684.54 ± 5.15
Orientin **	10.86 ± 0.25	15.30 ± 0.40	1578.22 ± 26.67	76.83 ± 0.82
Homoorientin **	11.53 ± 0.45	17.06 ± 0.56	1442.08 ± 24.04	75.38 ± 1.12
Vitexin **	-	-	54.58 ± 3.19	11.29 ± 1.57
Isovitexin**	-	-	37.63 ± 2.42	10.52 ± 0.76
Trolox *	2.97 ± 0.08	6.70 ± 0.16	250.16 ± 13.07	171.28 ± 2.93
Trolox **	11.87 ± 0.32	26.77 ± 0.64	999.48 ± 52.22	684.33 ± 11.71

Values are mean ± standard deviation of three replicate analyses. BSE, bamboo shavings extract; DF, Diethyl ether fraction; AF, acetic ether fraction; BF, *n*-butanol fraction; WF, water fraction. The antioxidant and free-radical scavenging activities are calculated based on dry weights of the bamboo shavings extract, the fractions and the characteristic compounds. *: µg /mL for IC_50_ of ABTS and DPPH assays, mg TEAC/g DW for TRAP assay and U/mg DW for total antioxidant capacity assay; **: µmol/L for IC_50_ of ABTS and DPPH assays, mg TEAC/ mmol DW for TRAP assay and U/µmol DW for total antioxidant capacity assay

**Table 4:** Correlation of total flavonoids (TF), total phenolics (TP), and total phenolic acids to antioxidant assay for bamboo shavings extract and its fractions.

Tests	TF	TP	Total Phenolic Acids
DPPH	0.925	0.854	0.844
ABTS	0.936	0.884	0.867
FRAP	0.979	0.890	0.945
TAC	0.909	0.741	0.887

IC_50_, IC_50_, reducing power and total antioxidant capacity of extracts were used to analysis the correlation index (R) with TF, TP and total phenolic acids on DPPH, ABTS, FRAP and TAC assay, respectively.

## Supplementary Materials

The following are available online at www.mdpi.com/1420-3049/21/8/996/s1, Figure S1: The RP-HPLC chromatograms of the (a) four flavone *C*-glucosides and fourphenolic acids standard mixture, (b) BSE, bamboo shavings extract; (c) DF, Diethylether fraction; (d) AF, acetic ether fraction; (e) BF, *n*-butanol fraction and (f) WF, water fraction all detected at 330 nm.Peak No.: (1) chlorogenic acid; (2) caffeic acid; (3) homoorientin; (4) orientin; (5) p-coumaric acid; (6) vitexin; (7) isovitexin; (8) ferulic acid.
