# Supplementary material for: Antioxidant Capacities of Fractions of Bamboo Shaving Extract and Their Antioxidant Components
Source: Molecules. 2016 Jul 30;21(8):996. doi: 10.3390/molecules21080996 (PMC6273639; doi:10.3390/molecules21080996)
Supplement: Supplementary file 1 [file molecules-21-00996-s001.pdf]

Supplementary Materials: Antioxidant Capacities of Fractions of Bamboo Shaving Extract and Their Antioxidant Components

Jinyan Gong, Jun Huang, Gongnian Xiao, Feng Chen, Bolim Lee, Qing Ge, Yuru You, Shiwang Liu and Ying Zhang

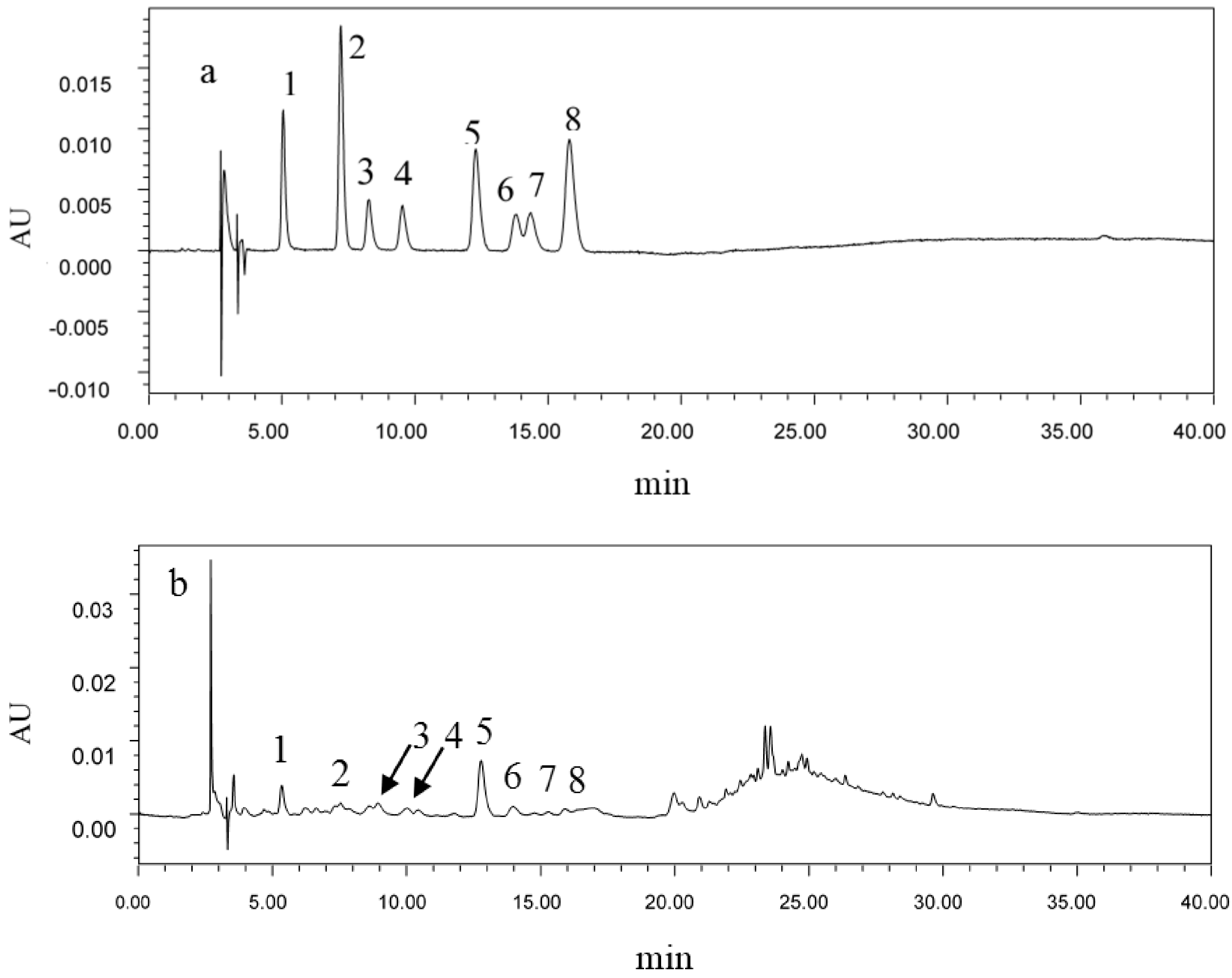

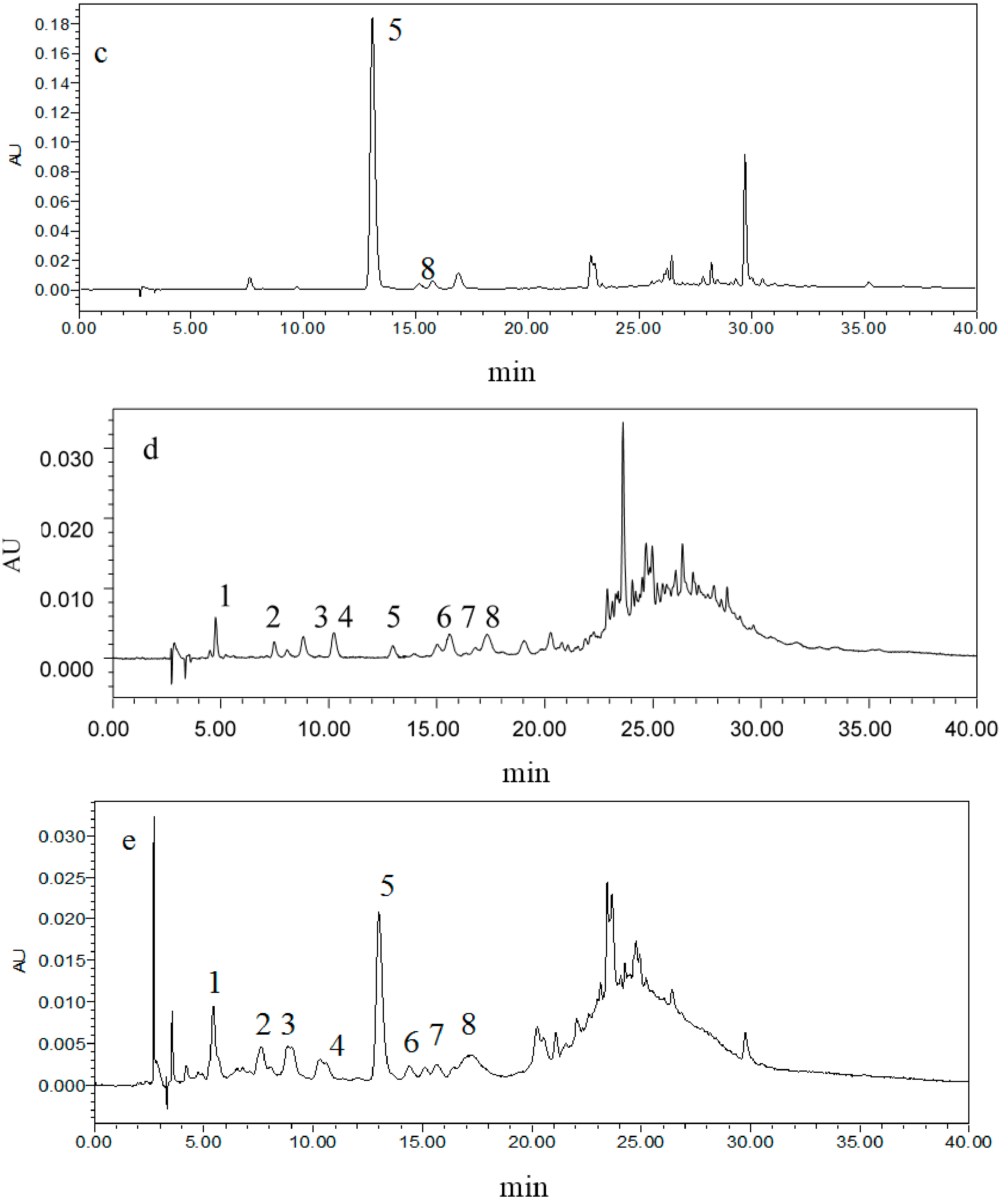
c
5
8
AU
0.18
0.16
0.14
0.12
0.10
0.08
0.06
0.04
0.02
0.00
0.00 5.00 10.00 15.00 20.00 25.00 30.00 35.00 40.00
min
d
1
2
3 4
5
6 7 8
AU
0.030
0.020
0.010
0.000
0.00 5.00 10.00 15.00 20.00 25.00 30.00 35.00 40.00
min
e
1
2 3
4
5
6 7
8
AU
0.030
0.025
0.020
0.015
0.010
0.005
0.000
0.00 5.00 10.00 15.00 20.00 25.00 30.00 35.00 40.00
min

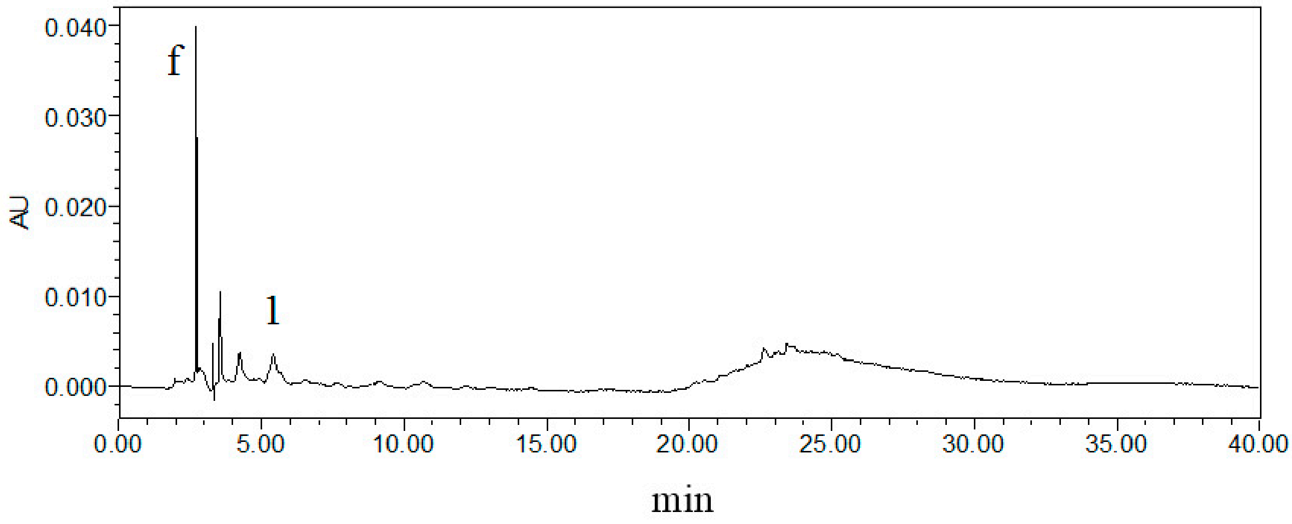

Figure S1. The RP-HPLC chromatograms of the (**a**) four flavone C-glucosides and fourphenolic acids standard mixture, (**b**) BSE, bamboo shavings extract; (**c**) DF, Diethylether fraction; (**d**) AF, acetic ether fraction; (**e**) BF, n-butanol fraction and (**f**) WF, water fraction all detected at 330 nm.Peak No.: (1) chlorogenic acid; (2) caffeic acid; (3) homoorientin; (4) orientin; (5) p-coumaric acid; (6) vitexin; (7) isovitexin; (8) ferulic acid.
